# Machine learning for ECG diagnosis and risk stratification of occlusion myocardial infarction

**DOI:** 10.1038/s41591-023-02396-3

**Published:** 2023-06-29

**Authors:** Salah S. Al-Zaiti, Christian Martin-Gill, Jessica K. Zègre-Hemsey, Zeineb Bouzid, Ziad Faramand, Mohammad O. Alrawashdeh, Richard E. Gregg, Stephanie Helman, Nathan T. Riek, Karina Kraevsky-Phillips, Gilles Clermont, Murat Akcakaya, Susan M. Sereika, Peter Van Dam, Stephen W. Smith, Yochai Birnbaum, Samir Saba, Ervin Sejdic, Clifton W. Callaway

**Affiliations:** 1 Department of Acute & Tertiary Care Nursing, University of Pittsburgh, Pittsburgh, PA USA; 2 Department of Emergency Medicine, University of Pittsburgh, Pittsburgh, PA USA; 3 Department of Electrical & Computer Engineering, University of Pittsburgh, Pittsburgh, PA USA; 4 Division of Cardiology, University of Pittsburgh, Pittsburgh, PA USA; 5 University of Pittsburgh Medical Center, Pittsburgh, PA USA; 6 School of Nursing, University of North Carolina at Chapel Hill, Chapel Hill, NC USA; 7 Department of Emergency Medicine, Northeast Georgia Health System, Gainesville, GA USA; 8 School of Nursing, Jordan University of Science and Technology, Irbid, Jordan; 9 Department of Population Medicine, Harvard Medical School and Harvard Pilgrim Health Care Institute, Boston, MA USA; 10 Advanced Algorithm Development Center, Philips Healthcare, Cambridge, MA USA; 11 Department of Critical Care Medicine, University of Pittsburgh, Pittsburgh, PA USA; 12 Division of Cardiology, University Medical Center Utrecht, Utrecht, The Netherlands; 13 Department of Emergency Medicine, Hennepin Healthcare, Minneapolis, MN USA; 14 Department of Emergency Medicine, University of Minnesota, Minneapolis, MN USA; 15 Division of Cardiology, Baylor College of Medicine, Houston, TX USA; 16 Department of Electrical & Computer Engineering, University of Toronto, Toronto, ON Canada; 17 Artificial Intelligence for Health Outcomes at Research & Innovation, North York General Hospital, Toronto, ON Canada

**Keywords:** Translational research, Myocardial infarction, Machine learning

## Abstract

Patients with occlusion myocardial infarction (OMI) and no ST-elevation on presenting electrocardiogram (ECG) are increasing in numbers. These patients have a poor prognosis and would benefit from immediate reperfusion therapy, but, currently, there are no accurate tools to identify them during initial triage. Here we report, to our knowledge, the first observational cohort study to develop machine learning models for the ECG diagnosis of OMI. Using 7,313 consecutive patients from multiple clinical sites, we derived and externally validated an intelligent model that outperformed practicing clinicians and other widely used commercial interpretation systems, substantially boosting both precision and sensitivity. Our derived OMI risk score provided enhanced rule-in and rule-out accuracy relevant to routine care, and, when combined with the clinical judgment of trained emergency personnel, it helped correctly reclassify one in three patients with chest pain. ECG features driving our models were validated by clinical experts, providing plausible mechanistic links to myocardial injury.

## Abstract (web summary)

A machine learning algorithm, developed to detect occlusion myocardial infarction with no-ST elevation from electrocardiogram, outperforms clinicians in diagnostic assessments.

## Main

**Extended Data Fig. 1:**
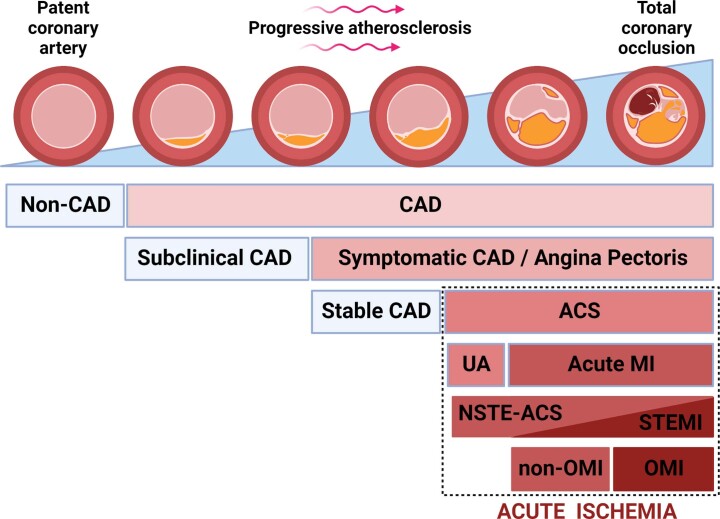
The relationship between the magnitude of vessel occlusion and the classification of acute coronary events. This figure shows the spectrum of coronary artery disease (CAD) as a function of severity and extent of atherosclerosis plaque progression, ranging from patent coronary artery (far left) to total coronary occlusion (far right). Among patients who develop symptomatic CAD, including those evaluated for chest pain or angina-like symptoms, a subset is diagnosed with acute coronary syndrome (ACS). This group is subclassified as either acute myocardial infarction (MI) or unstable angina (UA). Those with acute MI can be further subclassified, based on the presence of ST-elevation on the ECG, as either ST-elevation myocardial infarction (STEMI) or without ST-elevation (NSTEMI). The STEMI and NSTEMI patients overlap in terms of the presence or absence of total occlusion (depicted as triangles across the continuum in the figure). Alternatively, the same group with acute MI can be subclassified, based on angiographic TIMI flow criteria, as either occlusion (OMI) or non-occlusion (non-OMI) myocardial infarction. Unlike STEMI, OMI classification better aligns with focal angiographic findings since this group exclusively contains patients with total coronary occlusion. The color gradient indicates the severity of disease. This Figure was created with BioRender.com. Reproduced with permission from Al-Zaiti et. al.1 (permission number 5471421247333, Licensed content publisher: Elsevier).

The electrocardiogram (ECG) diagnosis of acute coronary syndrome (ACS) in patients with acute chest pain is a longstanding challenge in clinical practice^1–4^. Guidelines primarily focus on ST-segment elevation (STE) for discerning patients with ST-elevation myocardial infarction (STEMI) versus other forms of ACS^5–8^. A biomarker-driven approach is recommended in the absence of STE on the presenting ECG. This diagnostic paradigm has two important limitations. First, around 24–35% of patients with non-STEMI have total coronary occlusion, referred to as occlusion myocardial infarction (OMI), and require emergent catheterization^9–13^. This vulnerable group, in contrast to ACS with an open artery (Extended Data Fig. 1), suffers from unnecessary diagnostic and treatment delays that are associated with higher mortality^14–17^. This excess risk can be mitigated with enhanced diagnostic criteria. Although important ECG signatures of OMI are frequently described in the literature^18–21^, they are subtle, involve the entire QRST complex and are spatial in nature (that is, changes diluted across multiple leads)^22–24^. Visual inspection of ECG images by clinical experts is, thus, suboptimal and leads to a high degree of variability in ECG interpretation^25–27^.

The second limitation is that cardiac biomarkers, including conventional or high-sensitivity troponin (hs-cTn), cannot differentiate OMI until peak level is reached, which is too late to salvage myocardium. Positive troponin results (>99th percentile limit) come with a high false-positive rate, and approximately one-third of patients remain in a biomarker-indeterminate ‘observation zone’ after serial sampling^28,29^. More importantly, ~25% of acute myocardial infarction cases have a negative initial hs-cTn, which is observed in both the STEMI and OMI subgroups^30^. Consequently, 25–30% of patients with OMI are not treated in a timely fashion, and around 63% (interquartile range, 38–81%) of patients evaluated for chest pain at the emergency department are admitted to the hospital because of an inconclusive initial assessment^31^. These diagnostic limitations have created a costly, inefficient clinical practice paradigm where most patients with chest pain are over-monitored, whereas some patients with OMI have delayed diagnosis and treatment, potentially contributing to the 14–22% excess risk of mortality seen in the non-STE ACS (NSTE-ACS) group^15,32,33^.

**Extended Data Fig. 2:**
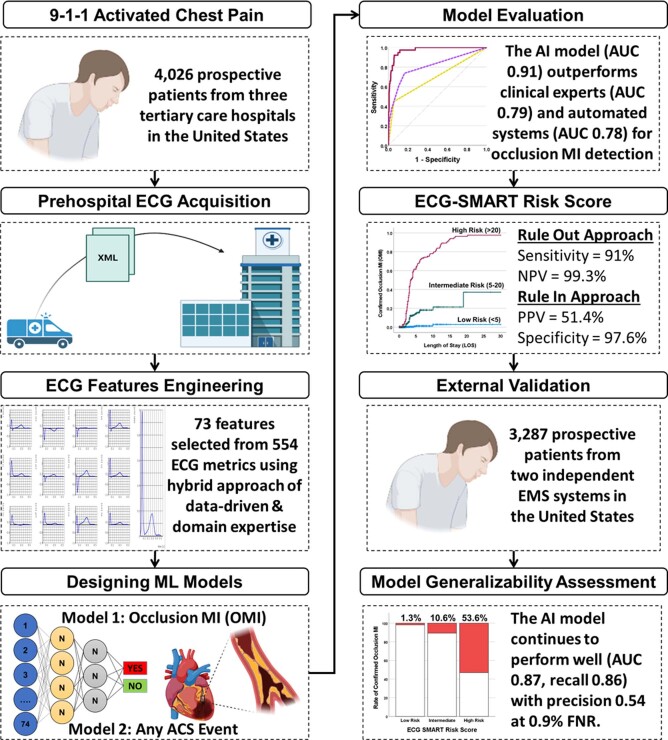
Graphical abstract summarizing the flow of study and main finding. This figure provides a graphical summary of the study flow and main findings. This Figure was created with BioRender.com (Credit to Salah Al-Zaiti).

In our previous work, we designed prototype algorithms for artificial intelligence (AI)-enabled ECG analysis and demonstrated the clinical feasibility of screening for ACS in the pre-hospital setting^34,35^. Here we describe, to our knowledge, the first multi-site, prospective, observational cohort study to evaluate the diagnostic accuracy of machine learning for the ECG diagnosis and risk stratification of OMI at first medical contact and in the absence of a STEMI pattern (Extended Data Fig. 2). Our intelligent models were derived and externally validated on 7,313 patients with chest pain from multiple clinical sites in the United States. The results demonstrate the superiority of machine learning in detecting subtle ischemic ECG changes indicative of OMI in the absence of a STEMI pattern, outperforming practicing clinicians and other widely used commercial ECG interpretation software. We identified the most important ECG features driving our model’s classifications and identified plausible mechanistic links to myocardial injury. Our derived OMI risk score provides enhanced rule-in and rule-out accuracy when compared to the HEART score, helping correctly reclassify one in three patients with chest pain. The benefits of this new clinical pathway in terms of clinical outcomes should be evaluated in prospective trials.

## Results

### Sample characteristics

**Fig. 1:**
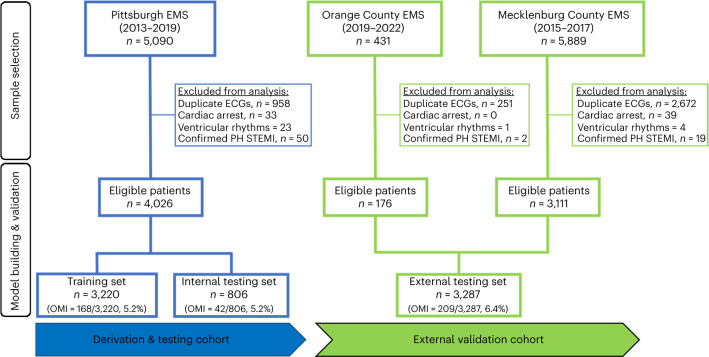
Cohort and sample selection. This flow diagram shows patient inclusion and exclusion criteria in each cohort as well as the dataset partition for training, internal testing and external validation cohorts. Exclusions are not mutually exclusive. EMS, Emergency Medical Services; PH, pre-hospital.

After excluding patients with cardiac arrest, ventricular tachyarrhythmias, confirmed pre-hospital STEMI and duplicate ECGs, our derivation cohort included 4,026 consecutive patients with chest pain (age 59 ± 16 years, 47% females, 5.2% OMI). The two external validation cohorts together included 3,287 patients (age 60 ± 15 years, 45% females, 6.4% OMI) (Fig. 1 and Table 1). Most patients in the derivation and validation cohorts were in normal sinus rhythm (>80%), and around 10% were in atrial fibrillation. Around 3% of patients had left bundle branch block (BBB), and ~10% had ECG evidence of left ventricular hypertrophy (LVH). The derivation and validation cohorts were similar in terms of age, sex, baseline clinical characteristics and 30-d cardiovascular mortality. The validation cohort, however, had more Black and Hispanic minorities and a slightly higher rate of ACS and OMI.

**Table 1:** Baseline demographic and clinical characteristics

	Derivation and testing cohort (*n* = 4,026)	External validation cohort (*n* = 3,287)
Age (years)	59 ± 16 (18−102)	60 ± 15 (21−100)
Sex		
Male	2,122 (53%)	1,814 (55%)
Female	1,904 (47%)	1,473 (45%)
Race		
White	1,698 (42%)	1,326 (40%)
Black	1,328 (33%)	1,544 (47%)
Others	52 (1.3%)	40 (1%)
Unknown	948 (24%)	377 (12%)
Ethnicity		
Not Hispanic	3,043 (76%)	2,850 (87%)
Hispanic / Latino	19 (1%)	116 (3.5%)
Unknown	964 (23%)	321 (9.5%)
Past medical history		
Hypertension	2,767 (69%)	2,090 (64%)
Diabetes	1,146 (29%)	1,067 (33%)
High cholesterol	1,520 (38%)	1,376 (42%)
Current smoker	1,244 (31%)	802 (25%)
Known CAD	1,388 (35%)	964 (30%)
Prior myocardial infarction	930 (23%)	929 (29%)
Prior PCI	963 (24%)	134 (4%)
Prior CABG	357 (10%)	470 (14%)
ECG and laboratory findings		
Sinus rhythm	3,496 (87%)	2,614 (80%)
Atrial fibrillation	354 (9%)	352 (11%)
Left BBB	94 (2.3%)	114 (3.5%)
Right BBB	237 (5.9%)	215 (6.6%)
ECG-LVH	383 (9.5%)	467 (14.2%)
cTnI positive (initial)	330 (8.2%)	736 (22.4%)
cTnI positive (serial testing)	729 (18.1%)	1,177 (35.8%)
Medical therapy		
PCI (any stent)	300 (7.5%)	245 (7.5%)
Emergent PCI (<90 min)	144 (3.6%)	157 (4.8%)
Total LAD occlusion	91 (2.3%)	94 (2.9%)
Total LCX occlusion	63 (1.6%)	88 (2.7%)
Total RCA occlusion	101 (2.5%)	102 (3.1%)
CABG	34 (0.8%)	30 (0.9%)
Study outcomes		
Confirmed ACS	550 (13.7%)	537 (16.3%)
OMI	210 (5.2%)	209 (6.4%)
Other acute myocardial infarction (NOMI)	240 (6.0%)	220 (6.7%)
Unstable angina	100 (2.5%)	108 (3.3%)
30-d cardiovascular death	137 (3.4%)	111 (3.4%)

Values are mean ± s.d. (minimum–maximum) or *n* (%). CABG, coronary artery bypass graft; NOMI: non-occlusion myocardial infarction; PCI: percutaneous coronary intervention.

### Algorithm derivation and testing

The positive class for model training was the presence of OMI, defined as a culprit coronary artery with a thrombolysis in myocardial infarction (TIMI) flow grade of 0–1, as adjudicated from charts by independent reviewers blinded to all ECG analyses. A TIMI flow grade of 2 with severe coronary narrowing (>70%) and peak fourth-generation (not high sensitivity) troponin of 5–10 ng ml^−1^ was also indicative of OMI. The negative class for model training was the absence of OMI, which included all other non-ACS etiologies or those with non-coronary occlusive ACS subtypes.

Input data for model training was based on pre-hospital 12-lead ECGs. We selected 73 morphological ECG features out of 554 temporal–spatial metrics using a hybrid data-driven and domain expertise approach^18^. Using these features, 10 classifiers were trained to learn ischemic patterns between ACS and non-ACS groups and to estimate the probability of OMI. We chose these classifiers to maximize the chance of finding the best-fitting approach for learning the mathematical representation relating complex ECG data to underlying physiology.

**Fig. 2:**
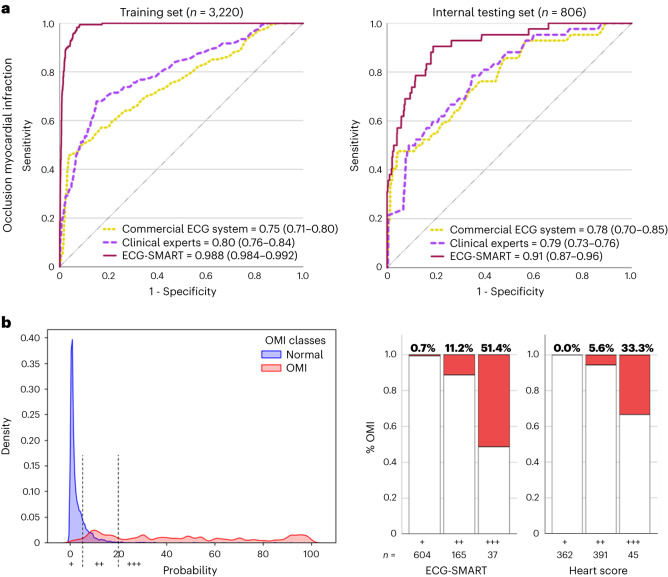
Algorithm derivation and testing. This figure shows the classification performance of the machine learning model against other reference standards for detecting OMI (**a**), the probability density plots of OMI(+) and OMI(−) classes as denoted by the machine learning model, along with optimal cutoffs of low risk, intermediate risk and high risk (**b**, **left**), and distribution of patients in low risk (+), intermediate risk (++) and high risk (+++) as per the machine learning model and HEART score (**b, right**).

The random forest (RF) model achieved the best bias–variance tradeoff for training and internal testing. We compared the RF against the ECG interpretation of practicing clinicians and against the performance of a commercial ECG interpretation system that is cleared by the US Food and Drug Administration (FDA) for ‘Acute MI’ diagnosis. On the hold-out test set, the RF model (area under the receiver operating characteristic (AUROC) 0.91 (95% confidence interval (CI) 0.87–0.96)) outperformed both practicing clinicians (AUROC 0.79 (95% CI 0.73–0.76), *P* < 0.001) and the commercial ECG system (AUROC 0.78 (95% CI 0.70–0.85), *P* < 0.001) (Fig. 2a).

Next, we used probability density plots for OMI(+) and OMI(−) classes to denote the optimal separation margins for risk prediction. As recommended by guidelines^6^, we defined a risk score to identify patients at low risk (OMI score <5), intermediate risk (OMI score 5–20) and high risk (OMI score >20), with these cutoffs yielding excellent separation between classes (log-rank chi-square, 133.04; degrees of freedom = 2; *P* < 0.001) (Fig. 2b, left). Our OMI score classified 74.4% of patients as low risk and 4.6% as high risk. Using the low-risk group in a rule-out strategy yielded a sensitivity of 0.91 and a negative predictive value (NPV) of 0.993, with an overall missed event rate of 0.5%. Using high-risk class for a rule-in strategy yielded a specificity of 0.976 and a positive predictive value (PPV) of 0.514, with an overall false discovery rate of 2%. Finally, we compared this OMI score to the HEART score, which uses patient history, ECG data, age, risk factors and troponin values (Fig. 2b, right). Our OMI score, which is based on ECG data alone, classified 66% more patients as low risk than the HEART score, with a similar false-negative rate <1%, and classified fewer patients as high risk and with much higher precision (51% versus 33%). The OMI score also triaged 50% fewer patients as intermediate risk and still got better discrimination for OMI detection (11.2% versus 5.6%).

### Model explainability

**Fig. 3:**
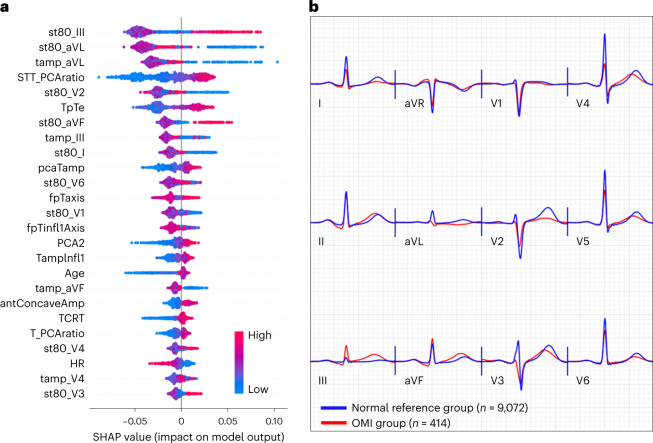
Model explainability for OMI detection. This figure shows SHAP values for the 25 most important features driving the predictions of the machine learning classifier in the derivation cohort (**a**) and the aggregate median beats of ECGs with OMI class (red) and the aggregate median beats of ECGs with normal sinus rhythm and no OMI (blue) (**b**). antConcaveAmp, the sum of concave amplitudes in the anterior leads; fpTaxis, T axis in the frontal plane; HR, heart rate; Infl1, the first inflection point before T peak; ST80, ST amplitude at the J point + 80 ms; tamp, T amplitude; TCRT, total cosine R-to-T; TpTe, T_peak_–T_end_ interval.

We used Tree SHAP algorithms to generate an importance ranking that explains the output of the RF model based on SHAP values estimated for the top 25 features (Fig. 3a). The features with the greatest impact on classification output included slight ST-depression in leads V1, V2, I and aVL; slight ST-elevation in leads III and V4–V6; loss of concave pattern in anterior leads; T wave enlargement in II and aVF and T flattening or inversion in I and aVL; prolonged T_peak_–T_end_ interval; T axis deviation; increased repolarization dispersion; and distorted directions of activation and recovery patterns. Most of these ECG patterns can be mechanistically linked to cardiac ischemia, suggesting their clinical value as plausible features for OMI detection.

To better visualize these global ECG patterns detected by our model, we created pooled population median beats for the OMI(+) class (*n* = 414 ECGs) and superimposed these median beats on the pooled population median beats of patients with normal sinus rhythm and OMI(–) status (*n* = 9,072 ECGs) (Fig. 3b). Findings from this figure support the patterns described by SHAP values above. Specifically, OMI is associated with ST-depression and T flattening in V1−V2, I and aVL; slight ST-elevation in the anterior leads with loss in concave pattern; peaked T wave in inferior leads; T_peak_ – T_end_ prolongation (seen in many leads); global repolarization dispersion (seen as peaked T in some leads and flattening in others); T axis deviation (away from the left ventricle); and distorted activation and recovery patterns (seen in the horizontal plane as loss of R wave progression in pre-cordial leads with increased T wave discordance). Due to prevalent multi-vessel disease in this cohort, these OMI patterns remained relatively consistent regardless of culprit location.

**Extended Data Fig. 3:**
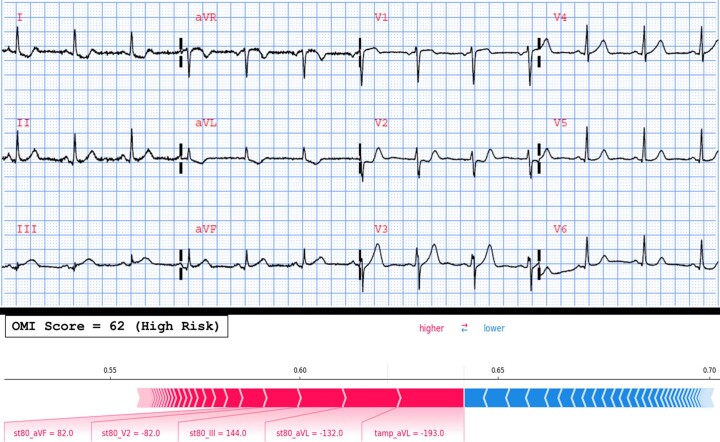
Local explainability of feature importance on a selected example. This figure shows the baseline ECG of a 50-year-old female with a past medical history of hypertension, high cholesterol, prior myocardial infarction, and current smoking. The ECG was documented as benign with isolated non-specific T wave changes, and the patient was triaged as intermediate risk. The patient was later sent to the catheterization lab where she had complete occlusion of the right coronary artery. The OMI score on this baseline ECG was 62 indicating high risk designation. The force plot identified the five most important ECG features that met the contribution threshold of the random forest model: negative T wave in aVL, slight ST depression in aVL and V2, and slight ST elevation in aVF and III.

Nevertheless, to examine local explainability of feature importance, we used force plots on individual cases to identify the features that met the contribution threshold of the RF model on a given ECG. These force plots were also examined by study investigators to further corroborate on the clinical validity of model predictions. Extended Data Fig. 3 shows a selected example of a 12-lead ECG with its corresponding force plot for the local features contribution.

### External validation

**Fig. 4:**
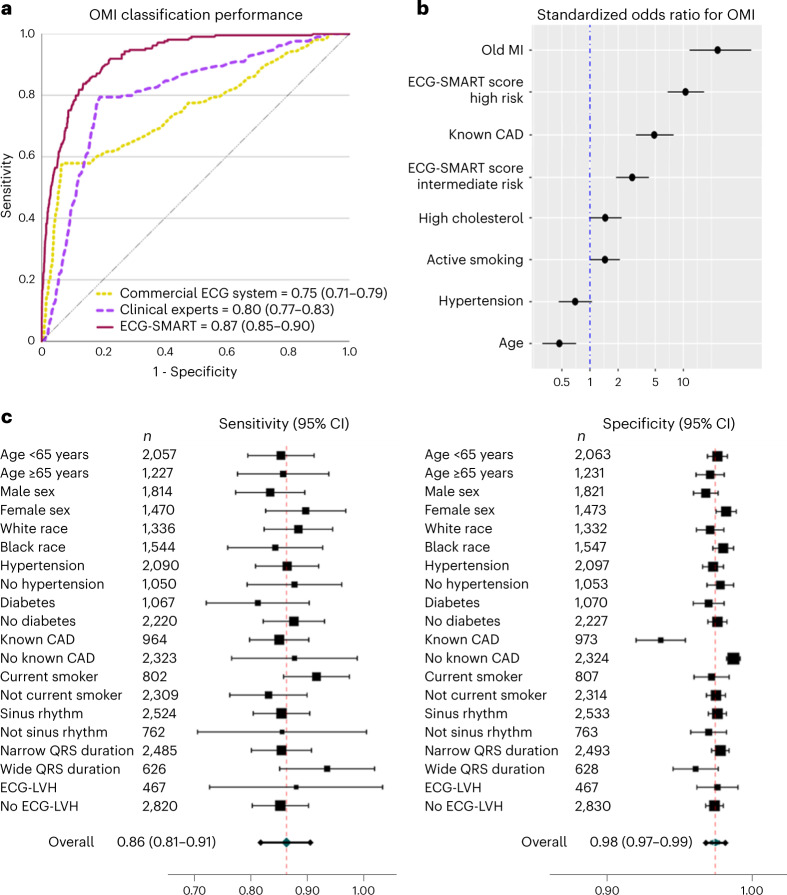
External validation of the ECG-SMART algorithm. **a**–**c**, This figure shows the classification performance of the machine learning model against other reference standards for detecting OMI on the external validation set (*n* = 3,287) (**a**), adjusted OR (center) with 95% CI (error bars) for the independent clinical predictors of OMI on the external validation set (*n* = 3,287) (**b**) and the overall sensitivity and specificity (center) with 95% CI (error bars) of the derived OMI score, along with breakdown across subgroups based on age, sex, comorbidities and baseline ECG findings (**c**). The size of the center marker is proportionate to the sample size of the respective subgroup.

We tested the final lock-out model on 3,287 patients from two independent external clinical sites. Machine learning engineers were blinded to outcome data from other sites, and the pre-populated model predictions were independently evaluated by the clinical investigators. Our model generalized well and maintained high classification performance (AUROC 0.87 (95% CI 0.85–0.90)), outperforming the commercial ECG system (AUROC 0.75 (95% CI 0.71–0.79), *P* < 0.001) and practicing clinicians (AUROC 0.80 (95% CI 0.77–0.83), *P* < 0.001) (Fig. 4a). Our OMI risk score was a strong predictor of OMI, independent from age, sex and other coronary risk factors (odds ratio (OR) 10.60 (95% CI 6.78–16.64) for high-risk class and OR 2.85 (95% CI 1.91–4.28) for intermediate-risk class) (Fig. 4b). This risk score triaged 69% of patients in the low-risk group at a false-negative rate of 1.3% and identified 5.1% of patients as high risk at acceptable true-positive rate >50%. The overall sensitivity, specificity, PPV and NPV for the OMI rule-in and rule-out strategy were 0.86 (95% CI 0.81–0.91), 0.98 (95% CI 0.97–0.99), 0.54 (95% CI 0.46–0.62) and 0.99 (95% CI 0.98–0.99), respectively. This diagnostic accuracy remained relatively similar across subgroups based on age, sex, race, comorbidities and baseline ECG findings, indicating the lack of aggregation bias (Fig. 4c). In comparison, the sensitivity, specificity, PPV and NPV for ECG overread by practicing clinicians were 0.58, 0.93, 0.36 and 0.97 and, for the commercial ECG system, 0.79, 0.80, 0.22 and 0.98, respectively.

**Fig. 5:**
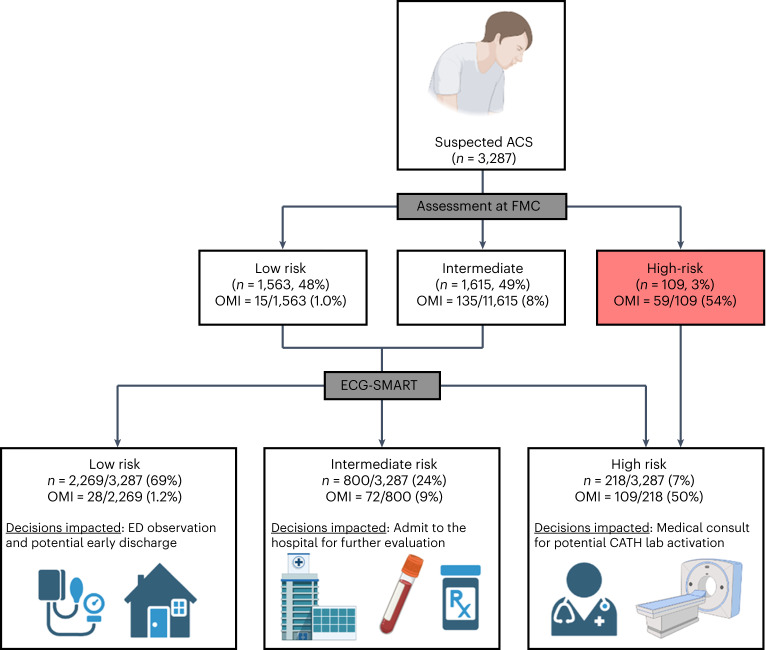
NRI of OMI risk score when integrated in the clinical workflow and concept of potential impact on subsequent clinical decisions. This figure describes the incremental gain of the derived risk score in reclassifying the initial triage decisions by emergency personnel at first medical contact and depicts the concept of potential impact on subsequent clinical decisions. This figure was created with BioRender (credit to S.S.A.-Z.). CATH, catheterization; ED, emergency department; FMC, first medical contact.

**Extended Data Fig. 4:**
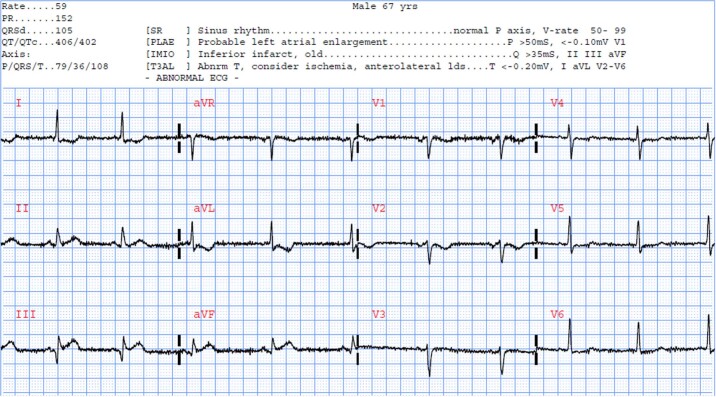
Selected example of a patient correctly reclassified as OMI. This figure shows an ECG that was correctly reclassified as occlusion myocardial infarction by the machine learning model. This baseline ECG was for a 67-year-old male with a past medical history of high cholesterol and a prior myocardial infarction. The ST-depression in anterior-lateral leads were noted, and the patient was triaged as intermediate risk. The OMI score was 49 indicating the need to up-triage. The patient was later sent to the catheterization lab where he had complete occlusion of the right coronary artery.

Next, we evaluated the incremental gain of our derived risk score in reclassifying patients at first medical contact (Fig. 5). Initial assessment by emergency personnel was based on the modified HEAR (history, ECG, age and risk factors) score to triage patients into low-risk, intermediate-risk and high-risk groups^36^. At baseline, emergency personnel triaged 48% of patients as low risk with an NPV of 99.0% and triaged 3% of patients as high risk with a PPV of 54.1%. Nearly 50% of patients remained in an indeterminate observation zone. Applying our OMI risk score would help triage 45% more patients as low risk while keeping the NPV at 98.8% and would help detect 85% more patients with OMI while keeping PPV at 50.0%. The OMI score would also help reduce the number of patients in the indeterminate observation zone by more than half. These numbers translate into a net reclassification improvement (NRI) index of 41% (95% CI 33–50%). To validate this incremental clinical utility, we manually reviewed ECGs reclassified correctly as OMI(+) (Extended Data Fig. 4). Many of these ECGs showed subtle or non-specific changes that were non-diagnostic as per guidelines^5^, suggesting potential value in boosting providers’ confidence when interpreting ‘fuzzy’ ECGs.

**Extended Data Fig. 5:**
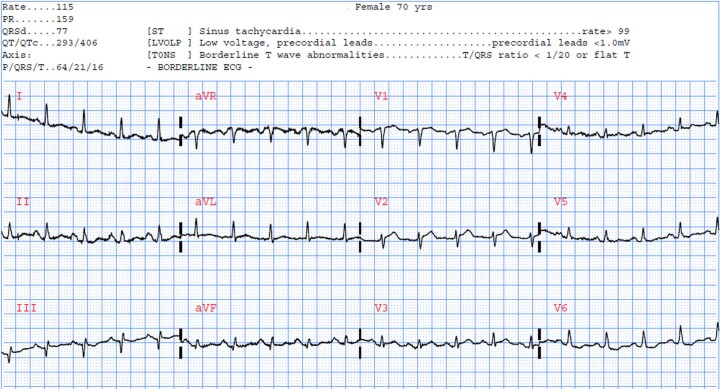
Selected example of a missed OMI by our model. This figure provides a selected example of a patient with occlusion myocardial infarction that was missed by the machine learning model and other reference standards. This ECG was obtained on a 70-year-old female with a past medical history of hypertension, high cholesterol, prior myocardial infarction, and current smoking. The baseline clinical interpretation suggests normal sinus rhythm with benign findings. There are isolated Q waves in inferior leads, low ECG voltage, and some baseline wander and high frequency noise in few leads. The OMI risk score was 2 indicating a low risk. The patient was later sent to the catheterization lab, which showed severe left main occlusion and had many stents placed. The patient developed new-onset HF during hospitalization. A closer look at this ECG by experienced ECG readers suggests that this ECG could resemble the ‘precordial swirl pattern’, a rightward ST-elevation vector, with STE in V1 and aVR and reciprocal ST-depression in V5 and V6. This pattern was found to correlate with LAD occlusion.

Finally, we investigated the potential sources of false negatives in the validation data. Among patients with missed OMI events (*n* = 28, 0.9%), many had high-frequency noise and baseline wander on their initial ECG (*n* = 13/28, 46%) or had low-voltage ECG (*n* = 14/28, 50%), and most patients (*n* = 24/28, 86%) had benign ECGs without any diagnostic ST-T changes (Extended Data Fig. 5). Moreover, we found no significant differences between false negatives and true positives in terms of demographics or clinical characteristics, with the exception that most false negatives had a history of a prior myocardial infarction (93% versus 27%). The latter finding was intriguing given that our OMI model was slightly less specific in patients with known coronary artery disease (CAD) (Fig. 4c). This finding aligns with recent evidence showing diminished NPV in patients with chest pain and known CAD^37^.

### Screening for any ACS event

**Extended Data Fig. 6:**
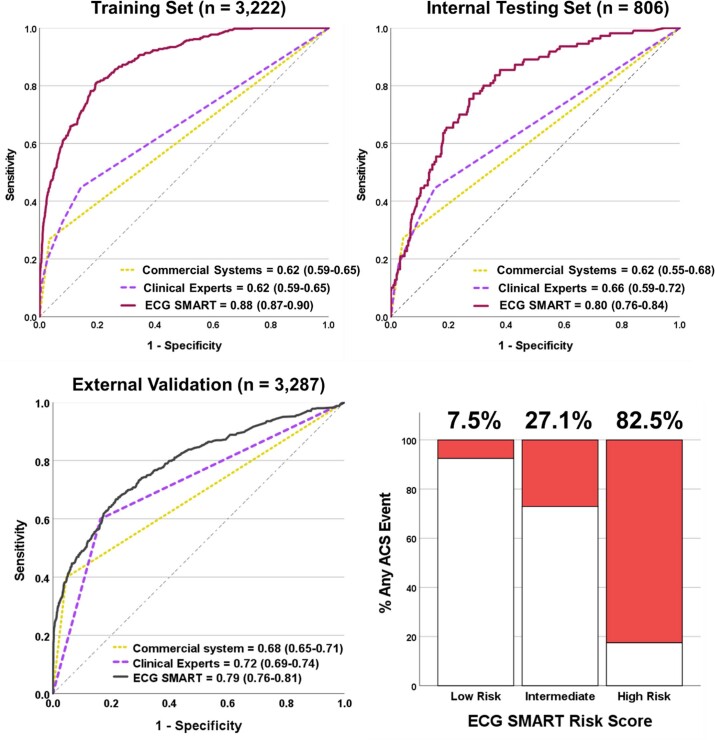
Development and validation of an algorithm to screen for any ACS event. This figure shows the classification performance of the machine learning model against other reference standards for detecting any acute coronary syndrome event (ACS). The figure also shows the distribution of patients in low-risk, intermediate risk, and high-risk groups as per our derived risk score. There is a notable gain in precision (rule-in) but a significant loss in recall (rule-out).

We further built a model to screen for any potential ACS event at first medical contact. Using the same set of ECG features, we trained and optimized an RF classifier that denoted the likelihood of any ACS event. The model performed well during training (AUROC 0.88 (95% CI 0.87–0.90)) and generalized well during internal testing (AUROC 0.80 (95% CI 0.76–0.84)) (Extended Data Fig. 6). On external validation, the model continued to generalize well (AUROC 0.79 (95% CI 0.76–0.8)), outperforming the commercial system (AUROC 0.68 (95% CI 0.65–0.71), *P* < 0.001) and practicing clinicians (AUROC 0.72 (95% CI 0.69–0.74), *P* < 0.001). Our derived risk score provided a suboptimal rule-out classification for any ACS event (sensitivity 68.2% and NPV 92.5%) but provided superior rule-in accuracy (specificity 98.9% and PPV 82.5%).

## Discussion

In this study, we developed and validated a machine learning algorithm for the ECG diagnosis of OMI in consecutive patients with chest pain recruited from multiple clinical sites in the United States. This model outperformed practicing clinicians and other commercial interpretation systems. The derived risk score provided superior rule-in and rule-out accuracy for OMI, boosting the sensitivity by ~28 percentage points and the precision by ~32 percentage points compared to reference standards. When combined with the judgment of experienced emergency personnel, our derived OMI risk score helped correctly reclassify one in three patients with chest pain. To our knowledge, this is the first study using machine learning methods and novel ECG features to optimize OMI detection in patients with acute chest pain and negative STEMI pattern on their presenting ECG.

Mapping myocardial ischemia, a problem of regional metabolic derangement, to coronary occlusion, a problem of diminished blood flow due to an atherosclerotic plaque rupture, is a complex process^1^. Essentially, ischemia disproportionately distorts action potentials in different myocardial segments, resulting in tissue-scale currents, often called ‘injury’ currents. Previous studies mapped pronounced ST-elevation to transmural injury currents associated with total coronary occlusion. This has historically driven the current paradigm dichotomy of STEMI versus ‘others’ (any ACS other than STEMI) in determining who might benefit from emergent reperfusion therapy. However, nearly 65% of patients with ACS present with no ST-elevation on their baseline ECG^35,38^, and, among the latter group, 24–35% have total coronary occlusion requiring emergent catheterization^9–13^. Thus, determining who would benefit from reperfusion therapy remains an adjudicated diagnosis.

**Extended Data Fig. 7:**
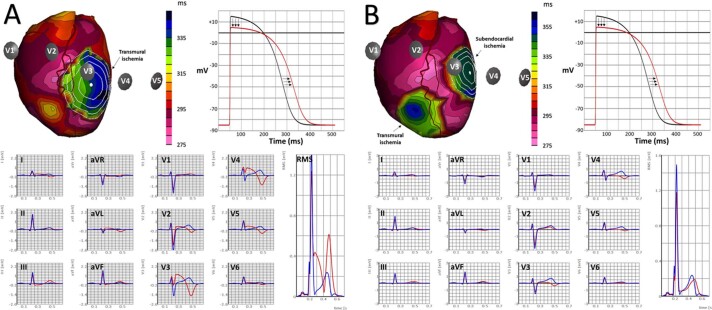
Limitations of ST amplitude on surface ECG as a sole marker of myocardial ischemia. This figure shows: (**a**) cardiac model of anterior wall epicardial ischemia with corresponding ST-elevation on V3 to V5 of the 12-lead ECG. (**b**) cardiac model of anterolateral and inferior-apical epicardial ischemia with corresponding attenuation of ST changes on the 12-lead ECG. This figure was generated using ECGSIM (www.ecgsim.org). Reproduced with permission from Al-Zaiti et. al.1 (permission number 5471421247333, Licensed content publisher: Elsevier).

Conceptually, injury currents produced by ischemic cardiac cells are summative in nature, explaining how ST amplitude changes can get attenuated on the surface ECG (Extended Data Fig. 7). These injury currents, however, distort the propagation of both excitation and recovery pathways, altering the configuration of the QRS complex and the ST-T waveform altogether^39^. Thus, a more comprehensive approach for the ECG detection of ischemia should focus on (1) evaluating temporal characteristics over entire waveform segments rather than the voltage at a given timepoint (for example, J point + 80 ms) and (2) evaluating lead-to-lead spatial characteristics in waveform morphology rather than absolute changes in isolated ECG leads^1^.

This study identified several ECG patterns indicative of acute coronary occlusion beyond the criteria recommended by clinical guidelines^5^. Intriguingly, these ECG patterns overlap with those described in the literature. A consensus report in 2012 identified few ECG patterns that should be treated as STEMI equivalent during acute pain episodes: ST-depression in V1–V3; small inverted T waves in V1–V3; deep negative T waves in pre-cordial leads; widespread ST-depression; and prominent positive T waves^20^. Similar ECG patterns were also described more recently: ST-depression in V1–V4 (versus V5–V6); reciprocal ST-depression with maximal ST-depression vector toward the apex (leads II and V5, with reciprocal STE in aVR); subtle ST-elevation; acute pathologic Q waves; hyperacute T waves; and loss of terminal S wave^21^. Many of these expert-driven patterns rely on assessing the proportion of repolarization amplitudes or area under the QRS amplitude. They also rely heavily on the visual assessment of waveform morphology and can introduce a high degree of subjectivity and variability among ECG interpreters. We demonstrated that machine learning models not only outperformed practicing clinicians in identifying OMI but also provided an objective, observer-independent approach to quantifying subtle ECG patterns associated with OMI.

Many of the data-driven features identified by our machine learning model are subtle and cannot be easily appreciated by clinical experts. T feature indices were among these most important features, including T_peak_–T_end_ interval prolongation, T wave flattening and T wave characteristics at the inflection point preceding T_peak_ (Fig. 3a). Mechanistically, ischemic injury currents interfere with signal propagation, leading to longer activation time^40^. These late activation potentials lead to a loss of terminal S wave and longer recovery time, both manifesting as T wave flattening, shifted T peak and loss of concavity at the initial T wave (Fig. 3b). These STEMI-equivalent patterns were previously described in the literature as small or negative T waves with widespread ST-depression or subtle ST-elevation^20,21^. Another important subtle feature identified by our model was increased ventricular repolarization dispersion, measured using the ratio between the principal components of the ST-T waveforms (that is, principal component analysis (PCA) metrics), the direction of the T axis and the angle between activation and recovery pathways (for example, total cosine R-to-T). Injury currents disproportionately affect the duration and velocity of repolarization across different myocardial segments^41^, resulting in lead-to-lead variability in the morphology of the ST-T waveform^22–24,39,42^. These high-risk ECG patterns were previously described as a mixture of deep negative T waves and prominent/hyperacute T waves or reciprocal T wave changes^20,21^. Our machine learning model provided a more comprehensive, quantitative approach to evaluating this subtle inter-lead variability in repolarization morphology.

Machine learning is well suited to address many challenges in 12-lead ECG interpretation. Myocardial ischemia distorts the duration and amplitude of the Q wave, R peak, R′, QRS complex, ST segment and T wave as well as the morphology and configuration of these waveforms (for example, upsloping, downsloping, concavity, symmetry and notching). These distortions are lead specific yet come with dynamic inter-lead correlations. Thus, ECG interpretation involves many complex aspects and parameters, making it a highly dimensional, decision space problem^1^. Few experienced clinicians excel in such pattern recognition,^21^ which explains why so many patients with OMI are not reperfused in a timely way; this is also why simple, rule-based commercial systems that use simple regression models are suboptimal for OMI detection. Machine learning algorithms can provide powerful tools to solve such highly dimensional, nonlinear mathematical representations found in 12-lead ECG data.

Although the literature on machine learning for the ECG diagnosis of coronary disease is ubiquitous, it comes with many serious limitations. First, many studies focused on detecting the known STEMI group^34,35,43,44^ rather than the critical OMI group without ST-elevation. Second, most previous work used open-source ECG datasets, such as PTB and PTB-XL^45^, which are highly selected datasets that focus on ECG-adjudicated diagnoses. Our unique cohorts included unselected, consecutive patients with clinical profiles and disease prevalence like that seen in real-world settings. Third, many studies used a full range of input features based on both ECG data and clinical data elements (for example, patient history, physical examination abnormalities, laboratory values and diagnostic tests)^46–49^, which limits the applicability to real-world settings. Fourth, to our knowledge, most studies used a single derivation cohort for training and testing^50^, without the use of an independent validation cohort. Finally, previous studies paid little attention to model explainability^51^, shedding little light on novel markers and pathways of ischemia than what is already known. Without explanation aids of clinical meaningfulness, machine learning models for ECG interpretation would have limited clinical utility^52^.

This study has important clinical implications. Our model can be integrated into systems of care for real-time deployment where risk score assignments can be made readily available to clinicians at the time of ECG acquisition. Enhanced decision support can help emergency personnel identify 85% more patients with critical coronary occlusion despite the absence of a STEMI pattern and without any loss in precision. Our models can also help inform care in more than 50% of patients in whom the initial assessment is indeterminate, placing 45% more patients in the low-risk group for OMI without any loss in NPV. This incremental gain in rule-in and rule-out accuracy can help re-allocate critical emergency resources to those in utmost need while optimizing the clinical workflow. This can impact numerous decisions at first medical contact, including targeted pre-hospital interventions, catheterization laboratory activation, administration of anti-ischemic therapies, hospital destination decisions, the need for medical consults, referrals for expedited diagnostic testing (for example, ECG and imaging scans) and early discharge decisions. Furthermore, until now, clinicians never had sensitive or highly specific tools that would allow the ultra-early identification of OMI in the absence of a STEMI pattern. Such enhanced diagnostics can allow the design and implementation of prospective interventional trials to assess the therapeutic effectiveness of targeted interventions in this vulnerable group (for example, early upstream P2Y_12_ inhibitor administration^53^, emergent versus delayed reperfusion therapy^54^ and glucose–insulin–potassium infusion^55^).

Several limitations merit consideration. First, the features that we used for model building were based on manufacturer-specific software. There are known discrepancies between manufacturers in ECG pre-processing, which means that our models would need retraining when using different software for signal processing. Alternatively, deep neural networks can be used to analyze raw ECG signal without explicit feature engineering. However, these techniques require much training samples (for example, >10,000) and might not yield a meaningful improvement over feature engineering-based machine learning for traditional 12-lead ECG-based diagnosis^56^. Second, we found slight differences between the derivation and validation cohorts in terms of disease prevalence and practicing clinicians’ accuracy in ECG interpretation. These cohorts came from two different regions in the United States, and emergency medical systems (EMSs) follow state-specific protocols. It is possible that discrepancies in EMS protocols and in-hospital practices resulted in slight differences in types and proportions of patients who receive pre-hospital 12-lead ECGs and the corresponding outcome adjudications. However, it is reassuring that our models continued to generalize well among the study sites. Third, it is worth noting that our model for ‘any ACS event’ boosted the performance of only the rule-in arm. This means that a low-risk determination suggests that a given patient would unlikely have OMI, but they might have a less subtle phenotype of NSTE-ACS that does not require reperfusion therapy. It is likely that serial ECG testing might improve the detection of missed events where patients might switch to a higher-risk category in the following hours^34^, but this remains to be confirmed. Coronary occlusion is a dynamic process that evolves over time, so an initial low-risk class by our models should not lead to a lower level of active monitoring. Finally, although this study used prospective patients, all analyses were completed offline. Prospective validation where OMI probabilities and decision support is provided in real time is warranted.

In conclusion, we developed and externally validated models for the ECG diagnosis of OMI in 7,313 patients with chest pain from multiple sites in the United States. The results demonstrated the superiority of machine learning in detecting subtle ischemic ECG changes indicative of OMI in an observer-independent approach. These models outperformed practicing clinicians and commercial ECG interpretation software, significantly boosting precision and recall. ECG features driving our models were evaluated, providing plausible mechanistic links to myocardial injury. Our derived OMI risk score provided enhanced rule-in and rule-out accuracy when compared to HEAR score, and, when combined with the clinical judgment of trained emergency personnel, this score helped correctly reclassify one in three patients with chest pain. The benefits of this new clinical pathway in terms of clinical outcomes should be evaluated in prospective trials. Future work should also focus on the prospective deployment where OMI probabilities and decision support is provided in real time.

## Methods

### Ethics statement

The derivation cohort included pre-hospital data from the City of Pittsburgh Bureau of Emergency Medical Services and in-hospital data from three tertiary care hospitals from the University of Pittsburgh Medical Center (UPMC) healthcare system: UPMC Presbyterian Hospital, UPMC Shadyside Hospital and UPMC Mercy Hospital (Pittsburgh, Pennsylvania). All consecutive eligible patients were recruited under a waiver of informed consent. This observational trial was approved by the institutional review board of the University of Pittsburgh and was registered at https://www.clinicaltrials.gov/ (identifier NCT04237688). The analyses described in this paper were pre-specified by the trial protocol that was funded by the National Institutes of Health. The first external validation cohort included data from Orange County Emergency Medical Services (Chapel Hill, North Carolina). This study actively consented eligible patients and was approved by the institutional review board of the University of North Carolina at Chapel Hill. The second external validation cohort included data from Mecklenburg County Emergency Medical Services and Atrium Health (Charlotte, North Carolina). Data were collected through a healthcare registry, and all consecutive eligible patients were enrolled under a waiver of informed consent. This study was also approved by the institutional review board of the University of North Carolina at Chapel Hill. These two external datasets were collected by the same local investigative team and were similar in terms of age, sex and disease prevalence. Thus, we combined these two datasets into one cohort for external validation purposes.

### Study design and data collection

This was a prospective, observational cohort study. The methods for each study cohort were described in detail elsewhere^57,58^. All study cohorts enrolled adult patients with an emergency call for non-traumatic chest pain or anginal equivalent symptoms (arm, shoulder or jaw pain, shortness of breath, diaphoresis or syncope). Eligible patients were transported by an ambulance and had at least one recorded pre-hospital 12‑lead ECG. There were no selective exclusion criteria based on sex, race, comorbidities or acuity of illness. For this pre-specified analysis, we included only non-duplicate ECGs from unique patient encounters, and we removed patients with pre-hospital ECGs showing ventricular tachycardia or ventricular fibrillation (that is, these patients are managed by ACLS algorithms). We also removed patients with confirmed pre-hospital STEMI, which included machine-generated ***ACUTE MI*** warning, EMS documentation of STEMI and medical consult for potential catheterization laboratory activation.

Independent reviewers extracted data elements from hospital systems on all patients meeting eligibility criteria. If a pre-hospital ECG had no patient identifiers, we used a probabilistic matching approach to link each encounter with the correct hospital record. This previously validated data linkage protocol was based on the ECG-stamped birth date, sex and date/time logs as well as based on EMS dispatch logs and receiving hospital records. All probabilistic matches were manually reviewed by research specialists for accuracy. The match success rate ranged from 98.6% to 99.8%.

### Clinical outcomes

Adjudications were made by independent reviewers at each local site after reviewing all available medical records within 30 d of the indexed encounter. Reviewers were blinded from all ECG analyses and models’ predictions. OMI was defined as coronary angiographic evidence of an acute culprit lesion in at least one of the three main coronary arteries (left anterior descending (LAD), left circumflex (LCX) and right coronary artery (RCA)) or their primary branches with TIMI flow grade of 0–1. TIMI flow grade of 2 with severe coronary narrowing >70% and peak troponin of 5–10.0 ng ml^−1^ was also considered indicative of OMI^17,21^. These adjudications were made by two independent reviewers. The kappa coefficient statistic between the two reviewers was 0.771 (that is, substantial agreement). All disagreements were resolved by a third reviewer.

ACS was defined per the Fourth Universal Definition of Myocardial Infarction as the presence of symptoms of ischemia (that is, diffuse discomfort in the chest, upper extremity, jaw or epigastric area for more than 20 min) and at least one of the following criteria: (1) subsequent development of labile, ischemic ECG changes (for example, ST changes and T inversion) during hospitalization; (2) elevation of cardiac troponin (that is, >99th percentile) during the hospital stay with rise and/or drop on serial testing; (3) coronary angiography demonstrating greater than 70% stenosis, with or without treatment; and/or (4) functional cardiac evaluation (stress testing) that demonstrates ECG, echocardiographic or radionuclide evidence of focal cardiac ischemia^5^. Patients with type 2 myocardial infarction and pre-existing subacute coronary occlusion were labeled as negative for ACS and OMI. This included around 10% of patients with positive troponin but with no rise and/or drop in concentration on serial testing (that is, chronic leak) or with troponin leak attributed to non-coronary occlusive conditions, such as pericarditis. On a randomly selected small subset of patients (*n* = 1,209), the kappa coefficient statistic for ACS adjudication ranged from 0.846 to 0.916 (that is, substantial to perfect agreement).

### ECG methods

Pre-hospital ECGs were obtained in the field by paramedics as part of routine care. ECGs were acquired using either Heart Start MRX (Philips Healthcare) or LIFEPAK-15 (Physio-Control) monitor–defibrillator devices. All digital 12-lead ECGs were acquired at a sampling rate of 500 samples per second (0.05–150 Hz) and transmitted to the respective EMS agency and receiving hospital. Digital ECG files were exported in .xml format and stored in a secondary server at each local site. ECG images were de-identified and manually annotated by independent reviewers or research specialists; ECGs with poor quality or missing leads were removed from the study. Next, digital .xml files were transmitted to the Philips Advanced Algorithm Research Center (Cambridge, Massachusetts) for offline analysis.

ECG featurization was described in detail elsewhere^18^. In brief, ECG signal pre-processing and feature extraction were performed using manufacturer-specific software (Philips DXL diagnostic 12/16 lead ECG analysis program). ECG signals were first pre-processed to remove noise, artifacts and baseline wander. Ectopic beats were removed, and representative median beats were calculated for each lead. Median beats refer to the representative average (or median) of the sequential beats in a given ECG lead after temporal alignment of R peaks. Next, we used the root mean square (RMS) signal to identify global waveform fiducials, including the onset, offset and peak of the P wave, QRS complex and T wave. Lead-specific fiducials were then identified to further segment individual waveforms into Q, R, R′, S, S′ and J point.

We then computed a total of 554 ECG features based on (1) the amplitude, duration, area, slope and/or concavity of global and lead-specific waveforms; (2) the QRS and T axes and angles in the frontal, horizontal, spatial, *x*–*y*, *x*–*z* and *y*–*z* planes, including directions at peak, inflection point and initial/terminal loops; (3) eigenvalues of the principal components of orthogonal ECG leads (I, II and V1–V6), including PCA ratios for individual ECG waveform segments; and (4) T loop morphology descriptors. Features with zero distribution were removed to prevent representation bias.

Next, we previously identified an optimal parsimonious list of the most important ECG features that are mechanistically linked to cardiac ischemia as described in detail elsewhere^18^. In brief, to prevent omitted feature bias, we used a hybrid approach that combines domain knowledge with a data-driven strategy. First, clinical scientists identified 24 classical features that are known to correlate with cardiac ischemia (that is, lead-specific ST and T wave amplitudes). Next, starting with a comprehensive list of 554 candidate features, we used data-driven algorithms (for example, recursive feature elimination and LASSO) to identify 198 supplemental features potentially related to ischemia. LASSO selects features with non-zero coefficients after L1 norm regularization, and recursive feature elimination uses repeated regression iterations to identify the features that have significant impact on model predictions. We then examined the feature pairs in this expanded list of 222 features and removed features with very high collinearity scores that contains redundant information (for example, we kept QTc if both QT and QTc were selected by the model). Finally, we used feature importance ranking to identify the most parsimonious subset of features that are complementary and can boost the classification performance. This hybrid approach eventually yielded a subset of 73 features that can serve as plausible markers of ischemia^18^.

### Machine learning methods

We followed best practices recommended by ‘ROBUST-ML’ and ‘ECG-AI stress test’ checklists to design and benchmark our machine learning algorithms^51,59^. To prevent measurement bias, ECG features were manually reviewed to identify erroneous calculations. Physiologically plausible outliers were replaced with ±3 s.d. On average, each feature had a 0.34% missingness rate (range, 0.1–1.6%). Thus, we imputed missing values with the mean, median or mode of that feature after consultation with clinical experts. ECG metrics were then *z*-score normalized and used as input features in machine learning models. The derivation and validation datasets were cleaned independently to prevent data leakage. Both cohorts were recruited over the same time window, suggesting the lack of temporal bias. To prevent potential mismatch with intended use, input features for model development included only ECG data plus the machine-stamped age. No other clinical data were used for model building.

We randomly split the derivation cohort into an 80% training set and a 20% internal testing set. On the training set, we fit 10 machine learning classifiers: regularized logistic regression, linear discriminant analysis, support vector machine (SVM), Gaussian naive Bayes, RF, gradient boosting machine, extreme gradient boosting, stochastic gradient descent logistic regression, *k*-nearest neighbors and artificial neural networks. Each classifier was optimized over 10-fold cross validation to fine-tune hyperparameters. After selecting optimal hyperparameters, models were retrained on the entire training subset to derive final weights and create a lockout model to evaluate on the hold-out test set. We calibrated our classifiers to produce a probabilistic output that can be interpreted as a confidence level (probability risk score). Trained models were compared using the AUROC curve with Wilcoxon signed-rank test for pairwise comparisons. ROC-optimized cutoffs were chosen using the Youden index, and classifications on confusion matrix were compared using McNemar’s test.

**Extended Data Fig. 8:**
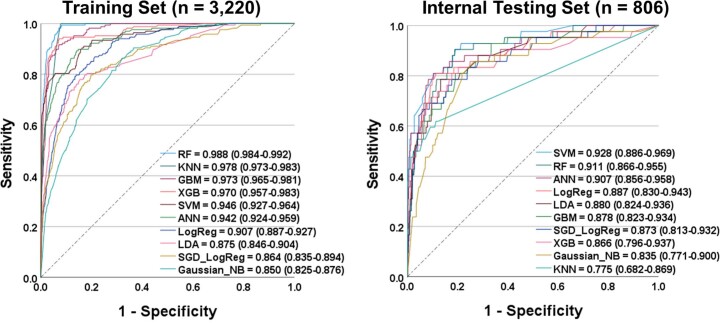
Comparison between 10 algorithms trained on the derivation cohort to classify OMI. This figure compares the area under the receiver operator characteristics curves (95% confidence interval) of 10 classifiers during training (left) and testing (right) on the derivation cohort. RF: random forest; KNN: K-nearest neighbors; GBM: gradient boosting machine; XGB: extreme gradient boosting; SVM: support vector machine; ANN: artificial neural networks; LogReg: regularized logistic regression; LDA: linear discriminant analysis; SGD_LogReg: stochastic gradient descent logistic regression; G_NB: Gaussian Naïve Bayes.

The RF classifier achieved high accuracy on the training set (low bias) with a relatively small drop in performance on the test set (low variance), indicating an acceptable bias–variance tradeoff and low risk of overfitting (Extended Data Fig. 8). Although the SVM model had lower variance on the test set, when compared to the RF model there were no significant differences in AUROC (Delong’s test) or their binary classifications (McNemar’s test). Moreover, there were no differences between the RF and SVM models in terms of Kolmogorov–Smirnov goodness of fit (0.716 versus 0.715) or the Gini purity index (0.82 versus 0.85). Due to its scalability and intuitive architecture, we chose the probability output of the RF model to build our derived OMI score. We generated density plots of these probability scores for positive and negative classes and selected classification thresholds for low-risk, intermediate-risk and high-risk groups based on pre-specified NPV > 0.99 and true-positive rate > 0.50. Finally, we used the lock-out RF classifier to generate probability scores and risk classes on the completely unseen external validation cohort. The code to generate probability scores is included with the supplementary materials of this manuscript.

### Reference standard

To reduce the risk of evaluation bias, we benchmarked our machine learning models against multiple reference standards used during routine care in clinical practice. First, we used a commercial, FDA-approved ECG interpretation software (Philips DXL diagnostic algorithm) to denote the likelihood of ischemic myocardial injury. This likelihood (yes/no) was based on a composite of the following: (1) diagnostic codes for ‘»>Acute MI«<’, including descriptive statements that denote ‘acute’, ‘recent’, ‘age indeterminate’, ‘possible’ or ‘probable’; and (2) diagnostic codes for ‘»>Acute Ischemia«<’, including descriptive statements that denote ‘possible’, ‘probable’ or ‘consider’. Diagnostic statements that denoted ‘old’ [infarct], ‘nonspecific’ [ST depression] or ‘secondary to’ [LVH or high heart rate] were excluded from this composite reference standard.

We also used practicing clinicians’ overread of ECGs to denote the likelihood of ischemic myocardial injury on a given ECG (yes/no) when a STEMI pattern does not exist, which is congruent with how emergency department physicians evaluate these patients in clinical practice. Independent physician reviewers annotated each 12-lead ECG image as per the Fourth Universal Definition of Myocardial Infarction criteria^5^, including two contiguous leads with ST-elevation (≥0.2 mV for V2–V3 in men ≥40 years of age and ≥2.5 mm in men <40 years of age; ≥0.15 mV for V2–V3 in women; or ≥0.1 mV in other leads) or ST-depression (new horizontal or downsloping depression ≥ 0.05 mV), with or without T wave inversion (>0.1 mV in leads with prominent R wave or R/S ratio > 1). Reviewers were also prompted to use their clinical judgment to identify highly suspicious ischemic changes (for example, reciprocal changes and hyperacute T waves) as well as to account for potential confounders (for example, BBBs and early repolarization). On a randomly selected subset of patients in the derivation cohort (*n* = 1,646), the kappa coefficient statistic between two emergency physicians who interpreted the ECGs was 0.568 (that is, moderate agreement). A third reviewer was used to adjudicate discrepancies on this randomly selected subset. Similarly, on a randomly selected subset of patients in the external validation cohort (*n* = 375), the kappa coefficient statistic between the two board-certified cardiologists who interpreted the ECGs was 0.690 (that is, substantial agreement).

Finally, given that clinicians largely depend on risk scores to triage patients in the absence of STEMI, which would greatly affect how patients with OMI are diagnosed and treated in clinical practice, we compared our derived OMI risk score against the HEART score. This score is commonly used in US hospitals, and it has been well validated for triaging patients in the emergency department^60^. The HEART score is based on the patient’s history at presentation, ECG interpretation, age, risk factors and initial troponin values (range, 0–10). This score places patients in low-risk (0–3), intermediate-risk (4–6) and high-risk (7–10) groups. Given that troponin results are not usually available at first medical contact, we used a modified HEAR score after dropping the troponin values, which has also been previously validated for use by paramedics before hospital arrival^36^. The comparison against the HEART score herein focused on establishing the incremental gain of using the derived OMI score over routine care at initial triage. We compared how the new risk classes assigned by our derived OMI score agree with or differ from the risk classes assigned by the HEART score, which could inform potential incremental gain over routine care.

### Statistical analysis

Descriptive statistics were reported as mean ± s.d. or *n* (%). Missing data were assessed for randomness and handled during ECG feature selection (see ‘Machine learning methods’ subsection above). Normality of distribution was assessed before hypothesis testing where deemed necessary. ECG features were *z*-score normalized as part of standard input architectures for machine learning models. Comparisons between cohorts were performed using the chi-square test (for discrete variables) and independent samples *t*-test or the Mann–Whitney *U*-test (for continuous variables). The level of significance was set at an alpha of 0.05 for two-tailed hypothesis testing where applicable.

All diagnostic accuracy values were reported as per Standards for Reporting Diagnostic Accuracy Studies (STARD) recommendations. We reported classification performance using AUROC curve, sensitivity (recall), specificity, PPV (precision) and NPV, along with 95% CI where applicable. For 10-fold cross validation, we compared the multiple classifiers using the Wilcoxon signed-rank test (for AUROC curves) and McNemar’s test (for confusion matrices). We derived low-risk, intermediate-risk and high-risk categories for the final classifier using kernel density plot estimates between classes. The adequacy of these risk classes was evaluated using log-rank chi-square of accumulative risk for clinically important outcomes over the length of stay during the indexed admission.

For assessing the incremental gain in classification performance, we compared the AUROC of the final model against reference standards using DeLong’s test. For ease of comparison, the confidence bounds for AUROC of the reference standards (commercial system and practicing clinicians) were generated using 1,000 bootstrap samples. To place the incremental gain value in a broader context of the clinical workflow, we also computed the NRI index of our model against the HEAR score during the initial assessment at first medical contact. Risk scores are an integral part of clinical workflow in patients with suspected ACS who do not meet STEMI criteria. As per STARD recommendations, the NRI index evaluates the net gain between up-triage and down-triage when correctly reclassifying risk class assignments of an ‘old’ test (HEART score) using a ‘new’ test (the derived OMI score).

We used logistic regression to identify the independent predictive value of OMI risk classes. We used variables significant in univariate analysis and then built multivariate models with the stepwise backward selection method using Wald chi-square criteria. We reported ORs with 95% CI for all significant predictors. All analyses were completed using Python version 3.8.5 and SPSS version 24.

### Reporting Summary

Further information on research design is available in the Nature Portfolio Reporting Summary linked to this article.

## Online content

Any methods, additional references, Nature Portfolio reporting summaries, source data, extended data, supplementary information, acknowledgements, peer review information; details of author contributions and competing interests; and statements of data and code availability are available at 10.1038/s41591-023-02396-3.

### Supplementary information

Reporting Summary

## Data Availability

The ECG-SMART trial makes use of extracted ECG features to train and evaluate an RF classifier to denote the probability of OMI. The ECG features used in the derivation and external validation datasets, along with linked clinical outcomes, are publicly available through GitHub (https://github.com/zeineb-bouzid/sharing-github-nature-medicine.git). Researchers wanting the source binary files to compute their own features should contact the corresponding author to arrange for proper approvals and institutional data use agreements. Interested researchers from non-commercial entities can submit a request by emailing the corresponding author at ssa33@pitt.edu. Requests will be processed within a 2-week timeframe.

## Code availability

The Python codes to evaluate these models, along with the derivation and external validation datasets, are available through GitHub (https://github.com/zeineb-bouzid/sharing-github-nature-medicine.git).
