# Supplementary material for: Machine learning for ECG diagnosis and risk stratification of occlusion myocardial infarction
Source: Nat Med. 2023 Jun 29;29(7):1804–13. doi: 10.1038/s41591-023-02396-3 (PMC10353937; doi:10.1038/s41591-023-02396-3)
Supplement: Supplementary file 1 — Reporting Summary [file 41591_2023_2396_MOESM1_ESM.pdf]

nature portfolio

Corresponding author(s): Salah Al-Zaiti

Last updated by author(s): May 1, 2023

Reporting Summary

Nature Portfolio wishes to improve the reproducibility of the work that we publish. This form provides structure for consistency and transparency in reporting. For further information on Nature Portfolio policies, see our Editorial Policies and the Editorial Policy Checklist.

Statistics

For all statistical analyses, confirm that the following items are present in the figure legend, table legend, main text, or Methods section.

n/a	Confirmed	
☐	☒	The exact sample size (*n*) for each experimental group/condition, given as a discrete number and unit of measurement
☐	☒	A statement on whether measurements were taken from distinct samples or whether the same sample was measured repeatedly
☐	☒	The statistical test(s) used AND whether they are one- or two-sided<br>*Only common tests should be described solely by name; describe more complex techniques in the Methods section.*
☐	☒	A description of all covariates tested
☐	☒	A description of any assumptions or corrections, such as tests of normality and adjustment for multiple comparisons
☐	☒	A full description of the statistical parameters including central tendency (e.g. means) or other basic estimates (e.g. regression coefficient) AND variation (e.g. standard deviation) or associated estimates of uncertainty (e.g. confidence intervals)
☐	☒	For null hypothesis testing, the test statistic (e.g. F, t, r) with confidence intervals, effect sizes, degrees of freedom and P value noted<br>*Give P values as exact values whenever suitable.*
☒	☐	For Bayesian analysis, information on the choice of priors and Markov chain Monte Carlo settings
☒	☐	For hierarchical and complex designs, identification of the appropriate level for tests and full reporting of outcomes
☒	☐	Estimates of effect sizes (e.g. Cohen's *d*, Pearson's *r*), indicating how they were calculated

Our web collection on statistics for biologists contains articles on many of the points above.

Software and code

Policy information about availability of computer code

Data collection	Prehospital 12-lead ECGs were obtained in the field by paramedics as part of routine care. These ECGs were acquired using either Heart Start MRX (Philips Healthcare) or LIFEPAK-15 (Physio-Control Inc.) monitor-defibrillator devices. Demographic and clinical data elements were abstracted from hospital systems on all patients meeting eligibility criteria. Adjudications of outcomes were made by independent reviewers at each local site after reviewing all available medical records within 30 days of the indexed encounter.
Data analysis	Each ECG was stored as a digital XML file, and all files were transmitted to the Philips Advanced Algorithm Research Center (Cambridge, Massachusetts, USA). These source binary files were preprocessed using the commercially available Philips DXL diagnostic 12/16 lead ECG analysis program. The extracted ECG features were then used for model derivation and testing. These ECG features are made available in CSV format along with the code used to derive the machine learning classifier. All diagnostic accuracy values were reported as per STARD recommendations (Reporting Guidelines for Diagnostic Accuracy Studies). We reported classification performance using AUROC curve, sensitivity (recall), specificity, PPV (precision), and NPV, along with 95% confidence interval (CI) where applicable. For 10-fold cross validation, we compared the multiple classifiers using the Wilcoxon signed-rank test (for AUROC curves) and McNemar's test (for confusion matrices). We derived low-, intermediate-, and high-risk categories for the final classifier using Kernel density plot estimates between classes. The adequacy of these risk classes was evaluated using Log-rank chi-square of accumulative risk for clinically important outcomes over the length of stay during the indexed admission. All analyses were completed using Python v3.8.5 and SPSS v24.

For manuscripts utilizing custom algorithms or software that are central to the research but not yet described in published literature, software must be made available to editors and reviewers. We strongly encourage code deposition in a community repository (e.g. GitHub). See the Nature Portfolio guidelines for submitting code & software for further information.

Data

Policy information about availability of data

All manuscripts must include a data availability statement. This statement should provide the following information, where applicable:

- Accession codes, unique identifiers, or web links for publicly available datasets
- A description of any restrictions on data availability
- For clinical datasets or third party data, please ensure that the statement adheres to our policy

The ECG SMART trial makes use of extracted ECG features to train and evaluate a random forest classifier to denote the probability of OMI. The ECG features used in the derivation and external validation datasets along with linked clinical outcomes are publicly available through GitHub (https://github.com/zeineb-bouzid/sharing-github-nature-medicine.git). Researchers wishing the source binary files to compute their own features should contact the corresponding author to arrange for proper approvals and institutional data use agreements. Interested researchers from non-commercial entities can submit a requested by emailing the corresponding author at ssa33@pitt.edu. Requests will be processed within 2-week time frame.

Human research participants

Policy information about studies involving human research participants and Sex and Gender in Research.

Reporting on sex and gender	Our manuscript is compliant with the Journal's policy on sex and gender reporting. As per the National Institute of Health, sex was treated as a biological variable in this study. We collected biological sex on all participants as documented in the electronic health records. We enrolled all consecutive patients with suspected acute coronary syndrome, so all eligible patients from both sexes were recruited. Given that the rate of outcomes is typically higher in males than females and to avoid algorithmic bias, we decided a priori not to include sex as an input feature to estimate probability of OMI in our models. Instead, we show post-hoc evaluation of the diagnostic accuracy of the OMI score in each sex separately (Fig. 4C). There were no significant differences in sensitivity and specificity of the OMI score between males and females.
Population characteristics	All study cohorts enrolled consecutive adult patients > 18 years of age with an emergency call for non-traumatic chest pain or anginal equivalent symptoms (arm, shoulder, jaw pain, shortness of breath, diaphoresis, syncope). Eligible patients were transported by an ambulance and had at least one recorded prehospital 12-lead ECG. There were no selective exclusion criteria based on sex, race, comorbidities, or acuity of illness. After excluding patients with cardiac arrest, ventricular tachyarrhythmias, confirmed prehospital STEMI, and duplicate ECGs, our derivation cohort included 4,026 consecutive patients with chest pain (age 59±16 years, 47% females, 5.2% OMI). The two external validation cohorts together included 3,287 patients (age 60±15 years, 45% females, 6.4% OMI). The derivation and validation cohorts were comparable in terms of age, sex, baseline clinical characteristics, and 30-day cardiovascular mortality.
Recruitment	This was an observational cohort study that enrolled all consecutive eligible patients. There were no selective exclusion criteria based on sex, race, comorbidities, or acuity of illness. All eligible, consecutive patients were recruited under a waiver of informed consent to minimize any potential sources of selection bias. We collected prehopsital ECGs obtained during routine care. Independent reviewers extracted data elements from hospital systems on all patients meeting eligibility criteria. Study cohorts altogether enrolled patients that were transported by a participating EMS agency between May 1, 2013, and June 29, 2022.
Ethics oversight	The derivation cohort included prehospital data from the City of Pittsburgh Bureau of Emergency Medical Services (EMS) and in-hospital data from the University of Pittsburgh Medical Center (UPMC). This cohort study was approved by the institutional review board of the University of Pittsburgh. The external validation cohorts included data from Orange County EMS and Mecklenburg County EMS and Atrium Health. These two cohort studies were approved by the institutional review board of the University of North Carolina at Chapel Hill.

Note that full information on the approval of the study protocol must also be provided in the manuscript.

Field-specific reporting

Please select the one below that is the best fit for your research. If you are not sure, read the appropriate sections before making your selection.

☒ Life sciences ☐ Behavioural & social sciences ☐ Ecological, evolutionary & environmental sciences

For a reference copy of the document with all sections, see nature.com/documents/nr-reporting-summary-flat.pdf

Life sciences study design

All studies must disclose on these points even when the disclosure is negative.

Sample size	No minimum sample size was specified. All eligible consecutive patients over the duration of the study were enrolled. All the available data for this analysis were used.

Data exclusions	We removed patients with confirmed prehospital ST-elevation myocardial infarction (STEMI) because these patients undergo emergent catheterization as per clinical guidelines without the need for additional diagnostic testing.
Replication	We used rigorous 10-fold cross-validation to estimate models' hyper-parameters on the derivation dataset and assessed bias-variance trade-off between training and internal testing splits. We also used an external validation cohort from two independent sites and demonstrated model generalizability on unseen patients. The results were reproducible across folds and datasets. We have made our Python code and the derivation and external validation datasets available to ensure reproducibility.
Randomization	Randomization was not relevant to this analysis. This was an observational cohort study and there was no active assignment of participants to groups.
Blinding	Blinding was not relevant to this analysis. This was an observational cohort study and there was no active assignment of participants to groups that required blinding..

Reporting for specific materials, systems and methods

We require information from authors about some types of materials, experimental systems and methods used in many studies. Here, indicate whether each material, system or method listed is relevant to your study. If you are not sure if a list item applies to your research, read the appropriate section before selecting a response.

Materials & experimental systems

n/a	Involved in the study	
☒	☐	Antibodies
☒	☐	Eukaryotic cell lines
☒	☐	Palaeontology and archaeology
☒	☐	Animals and other organisms
☐	☒	Clinical data
☒	☐	Dual use research of concern

Methods

n/a	Involved in the study	
☒	☐	ChIP-seq
☒	☐	Flow cytometry
☒	☐	MRI-based neuroimaging

Clinical data

Policy information about clinical studies

All manuscripts should comply with the ICMJE guidelines for publication of clinical research and a completed CONSORT checklist must be included with all submissions.

Clinical trial registration	This study was registered in www.ClinicalTrials.gov (identifier # NCT04237688).
Study protocol	The analyses described in this paper were pre-specified by the trial protocol that was funded by the National Institute of Health.
Data collection	This was an observational cohort study that enrolled all consecutive eligible patients. We collected prehopsital ECGs obtained during routine care. Independent reviewers extracted data elements from hospital systems on all patients meeting eligibility criteria. The derivation cohort included prehospital data from the City of Pittsburgh Bureau of Emergency Medical Services (EMS) and in-hospital data from the University of Pittsburgh Medical Center (UPMC). The external validation cohorts included data from Orange County EMS and Mecklenburg County EMS and Atrium Health. Study cohorts altogether enrolled patients that were transported by a participating EMS agency between May 1, 2013, and June 29, 2022. A brief synopsis of the study protocol can be found on the public domain of the National Institute of Health through www.NIHreporter.gov
Outcomes	The primary study outcomes were ACS and OMI, which were predefined on study protocol and ClinicalTrials.gov prior to start of the study. ACS was defined per the fourth universal definition of myocardial infarction as the presence of symptoms of ischemia (i.e. diffuse discomfort in the chest, upper extremity, jaw, or epigastric area for more than 20 minutes) and at least one of the following criteria: (1) subsequent development of labile, ischemic ECG changes (e.g., ST changes, T inversion) during hospitalization; (2) elevation of cardiac troponin (i.e., > 99th percentile) during the hospital stay with rise and/or drop on serial testing; (3) coronary angiography demonstrating greater than 70% stenosis, with or without treatment; and/or (4) functional cardiac evaluation (stress testing) that demonstrates ECG, echocardiographic, or radionuclide evidence of focal cardiac ischemia.<br>OMI was defined as coronary angiographic evidence of an acute culprit lesion in at least one of the three main coronary arteries (LAD, LCX, RCA) or their primary branches with TIMI flow grade of 0-1. TIMI flow grade of 2 with significant coronary narrowing > 70% and peak troponin of 5-10.0 ng/mL was also considered indicative of OMI.<br>Adjudications were made by independent reviewers at each local site after reviewing all available medical records within 30 days of the indexed encounter. Reviewers were blinded from all ECG analyses and models' predictions. Each outcome was adjudicated by two independent reviewers. The Kappa coefficient statistic between the two reviewers for OMI was 0.771 (i.e., substantial agreement) and for ACS was 0.846 to 0.916 (i.e., substantial to perfect agreement). All disagreements were resolved by a third reviewer.
